# Comparison of Plaque Removal Efficacy of a Novel Flossing Agent with the Conventional Floss: A Clinical Study

**DOI:** 10.5005/jp-journals-10005-1560

**Published:** 2018

**Authors:** Shital DP Kiran, Komal Ghiya, Disha Makwani, Rohan Bhatt, Megha Patel, Mohit Srivastava

**Affiliations:** 1-6 Department of Pedodontics and Preventive Dentistry, Karnavathi School of Dentistry, Uvarsad, Gujarat, India

**Keywords:** Gingivitis, Gumchucks, Oral hygiene

## Abstract

**Introduction:**

The various methods for plaque control include mechanical plaque control methods which comprises use of toothbrushes, flosses, interdental brushes, and chemical plaque control which includes mouthwashes, dentrifices. The need for the study was to prove the efficacy of flossing in children using gumchucks.

**Materials and methods:**

A total sample size of 24 children age groups 6–12 years according to chronological age were selected. In 12 patients, flossing using gumchucks was done and in 12 patients flossing using unwaxed floss without handle was done. Proximal plaque index was taken at 0,2,4,6 weeks to assess the efficacy of both types of floss in removal of interproximal plaque. At the end of 6 weeks, patient's parents were asked to fill up the questionnaire.

**Results:**

In the intragroup comparison for gumchucks, significant plaque reductions were found at 4 and 6 weeks. In the intragroup comparison for unwaxed floss, significant reduction for plaque marginal index were recorded from baseline to 2 and 4 weeks. In the intergroup comparison, significant reduction in plaque index was recorded at 4 and 6 weeks.

**Conclusion:**

Gumchucks have the high efficacy of plaque removal as well as easy in use for children routinely. Also when surveyed majority of the patents preferred gumchucks if available in the stores.

**How to cite this article:**

Kiran SDP, Ghiya K, Makwani D, Bhatt R, Patel M, Srivastava M. Comparison of Plaque Removal Efficacy of a Novel Flossing Agent with the Conventional Floss: A Clinical Study. Int J Clin Pediatr Dent, 2018;11(6):474-478

## INTRODUCTION

The presence of bacterial plaque is associated with the development of teeth.^1^ The causes of gingivitis, dental caries, and periodontal disease are initiated by colonization and accumulation of plaque.

The various methods for plaque control include mechanical plaque control methods which comprise the use of toothbrushes, flosses, interdental brushes and chemical plaque control which includes mouthwashes and dentrifices.^2^ Plaque removal ultimately leads to the reduction of severity of the oral disease. Utilizing a toothbrush to mechanically remove plaque is extremely effective yet is not capable of thorough removal when used alone.^3^ Research shows that a combination of both supra and subgingival plaque removal is important in reducing the onset and severity of the gingival disease.^4^

The use of dental floss as an aide to tooth brushing provides a plaque-removal benefit above that of tooth brushing alone.^5^ Routine dental flossing has been found to be astonishingly low. Dental floss is advised as an adjunct to tooth brushing for control of plaque and prevention of dental disease. Waxed and unwaxed floss are both being recommended; it has been suggested that the latter is superior.^6^

The primary problem associated with flossing is the patient's inability to perform flossing on a regular basis as part of daily oral hygiene.^7^ The various kinds of flosses are unbonded dental floss, bonded dental floss, bonded or unbonded floss with a drug additive intended to provide a beneficial prophylactic effect.^8^ This study was conducted to test the efficacy of plaque removal of a novel flossing agent exclusively designed for children.

## MATERIALS AND METHODS

The samples of children aged between 6 years and 12 years, both male and female, from Department of Pedodontics and Preventive dentistry outpatient department (OPD) of Karnavati School of Dentistry, Gandhinagar were enrolled. The final sample size was 24, which were based upon the power of 95 and confidence interval 95%. The protocol for this research was approved by the institutional ethical committee.

Subjects are informed and written parental consent for the same is obtained from each subject's parent.

### Inclusion Criteria

- Subjects of age group 6–12 years, both male and female and who are accompanied by the parent.
- Children who had an immunization schedule as per Indian Academy of Pediatrics.

### Exclusion Criteria

- Subjects who had received preventive oral prophylaxis within the previous month.
- Subjects who had a history of dental flossing one or more times per day regularly.
- Subjects, who had gross dental caries and/or oral hygiene neglect, exhibited advanced periodontitis or received active periodontal therapy within the previous 6 weeks.
- Medically compromised subjects were excluded.

**Fig. 1:**
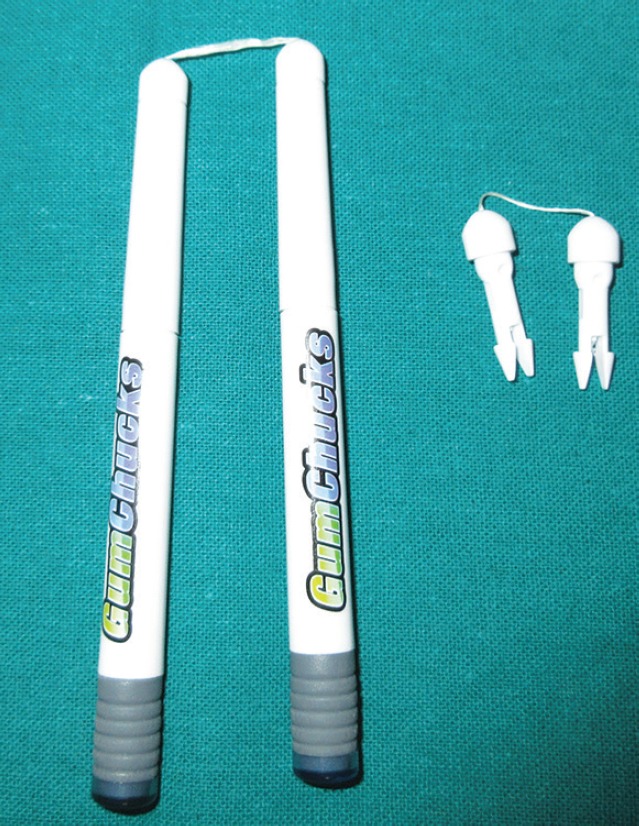
Gumchucks resembling like miniature nunchucks featuring disposable tips equipped with a 3/4-inch piece of dental floss

After considering inclusion and exclusion criteria total sample size of 24 children of age group 6–12 years according to chronological age were selected. In the first visit, the subjects in the study were selected and all the subjects were advised not to brush for 48 hours before the next visit. In 12 patients, flossing using gumchucks (Fig. 1) was done and in another 12 patients, flossing using unwaxed floss without a handle was performed.

Randomization of the sample was done by www.graphspad.com. Proximal plaque index (Table 1)^9^ was taken at 0, 2, 4 and 6 weeks to assess the efficacy of both types of floss in the removal of interproximal plaque. The patient in the last visit was asked to fill the questionnaire to assess the preference of flossing (Table 2) and the questions were based on patient satisfaction and marketing.

**Table 1:** Plaque marginal index (Benson et al.)^9^

0	No plaque
1	Separate flecks of plaque covering less than 1/3rd of the area
2	Discrete areas or bands of plaque covering less than 1/3rd of the area
3	Plaque covering 1/3rd of the area
4	Plaque covering more than 1/3rd but less than 2/3rd of the area
5	Plaque covering 2/3rd or more of the area

## RESULTS

From baseline to 6 weeks, the mean difference was −0.82 and −1.56. From 0 to 4 weeks, both the values indicated a significant reduction in the plaque index. Similarly, for 0 to 6 weeks, the mean difference was −2.53 which indicated significance, the plaque reduction. At 2 weeks and 4 weeks comparison, the plaque index was −0.74 which also indicates significant value.

When compared 2 and 6 weeks and 4 and 6 weeks, mean difference noted was −1.71 and −0.97, respectively, which also implies significance for intragroup comparison of gumchucks for the plaque index 0 to 2 weeks the *p* value was 0.002 whereas for all other week's *p* value was <0.001 (Table 3). From baseline to 2 weeks and baseline to 4 weeks intragroup comparison of unwanted floss for plaque index, the mean difference observed was −0.31 and 0.40, respectively. The *p* value noted was 0.015, 0.016, respectively, which indicates significance. Even the mean difference noted between 0 weeks to 6 weeks was −0.50 and the *p* value noted was 0.087 which imply significance (Table 4).

Intragroup comparison between gumchucks and unwaxed floss for plaque index at 0 weeks and 2 weeks, the mean difference between gumchucks and unwaxed floss was 0.26 and −0.25, respectively. Similarly, at 4 weeks and 6 weeks, the mean difference was −0.84 and −1.73, respectively. The *p* value at 4 weeks was 0.009 and 6 weeks it was < 0.001, which indicated significant changes in the plaque index (Table 5).

**Graph 1:**
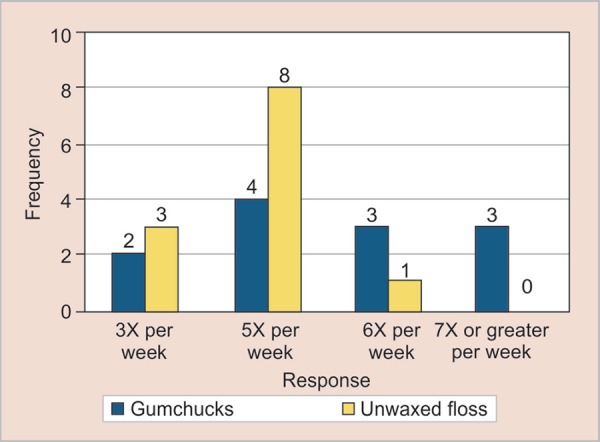
Questionnaire 1 results for how often does the child now flosses since the beginning of study

**Graph 2:**
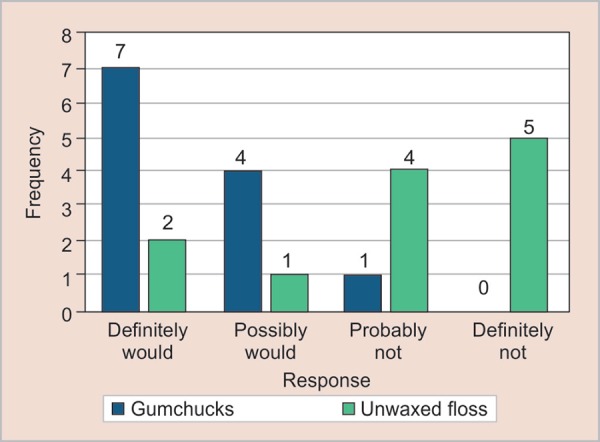
Questionnaire 2 results for would the parents purchase gumchucks if they were available in store

When we asked about (since the study began) how often does the child now flosses; 50% of the subjects have used 5 times per week, 12.5% parents have chosen for 7 times or greater per week, and 20.8% and 16.7% of the subjects answered that 3 times and 6 times, respectively (Graph 1). Therefore, overall the patients’ parents preferred to purchase gumchucks which appeared to be significant 0.0137 (Graph 2).

When questioned about if gumchucks/unwaxed floss were available in the store would they prefer to purchase it, 37.5% had answered yes and 20.8% had responded possibly would, probably not and definitely not (Graph 2). The *p* value noted was 0.010 which were significant, indicating a positive attitude towards acquiring gumchucks (Table 6).

**Table 2:** Questionnaire

Q1. Since the study began, how often does the child now flosses?	
(Fill in the blank)	
3X per week	□
5X per week	□
6X per week	□
7X or greater per week	□
Q2. If Gumchucks were available in the store, would you purchase it?	
Definitely would	□
Possibly would	□
Probably not	□
Definitely not	□

**Table 3:** Intragroup comparison of gumchucks for plaque index

	*Mean*	*Std. deviation*	*Std. error mean*	*Mean Difference*	*p value*
0 Weeks	6.69	1.18	0.340		
2 Weeks	5.87	0.76	0.220	−0.82	0.002
0 Weeks	6.69	1.18	0.340		
4 Weeks	5.13	0.85	0.245	−1.56	0.001
0 Weeks	6.69	1.18	0.340		
6 Weeks	4.16	0.84	0.244	−2.53	0.001
2 Weeks	5.87	0.76	0.220		
4 Weeks	5.13	0.85	0.245	−0.74	0.001
2 Weeks	5.87	0.76	0.220		
6 Weeks	4.16	0.84	0.244	−1.71	0.001
4 Weeks	5.13	0.85	0.245		
6 Weeks	4.16	0.84	0.244	−0.97	0.001

**Table 4:** Intragroup comparison of unwaxed floss for plaque index

	*Mean*	*Std. deviation*	*Std. error mean*	*Mean Difference*	*p value*
0 Weeks	6.43	0.54	0.156		
2 Weeks	6.12	0.45	0.130	−0.31	0.015
0 Weeks	6.38	0.54	0.162		
4 Weeks	5.97	0.50	0.151	−0.40	0.016
0 Weeks	6.39	0.56	0.177		
6 Weeks	5.89	0.68	0.214	−0.50	0.087
2 Weeks	6.10	0.47	0.140		
4 Weeks	5.97	0.50	0.151	−0.12	0.257
2 Weeks	6.13	0.48	0.152		
6 Weeks	5.89	0.68	0.214	−0.24	0.324
4 Weeks	5.97	0.53	0.167		
6 Weeks	4.16	0.84	0.244	−0.08	0.664

**Table 5:** Intergroup comparison between gumchucks and unwaxed floss for plaque index

*Group*		*Mean*	*Std. deviation*	*Std. error mean*	*Mean difference*	*p value*
0 Weeks	Gumchucks	6.69	1.18	0.340	0.26	0.488
	Unwaxed floss	6.43	0.54	0.156		
2 Weeks	Gumchucks	5.87	0.76	0.220	−0.25	0.338
	Unwaxed floss	6.12	0.45	0.130		
4 Weeks	Gumchucks	5.13	0.85	0.245	−0.84	0.009
		5.97	0.50	0.151		
6 Weeks	Gumchucks	4.16	0.84	0.244	-1.73	0.001
	Unwaxed floss	5.89	0.68	0.214		

## DISCUSSION

Plaque control is fundamental to the meaningful practice of preventive dentistry. Many investigators have validated that professionally administered prophylaxis or self-application of the toothbrush, dental floss, and other interdental devices efficiently remove interproximal plaque and reduce plaque scores, gingival inflammation and bleeding leading to generally improved gingival health.^10–12^ Starkey suggests that parents continue to brush for the child until the child has demonstrated efficiency which may be as late as 9–10 years.^13^ The subjects that were selected in the study were of the age 6–12 years, because below 6 years primary teeth have physiological spacing; therefore, flossing is not required, while in mixed dentition during age 6–12 years, these spaces are not present so for interproximal plaque removal flossing is required.

**Table 6:** Questionnaire results of the total count within the gumchucks and unwaxed groups

*Questions*		*Total count (%)*	*p value*
Question 1	3X per week	20.8	0.137
	5X per week	50.0	
	6X per week	16.7	
	7X or greater per week	12.5	
Question 2	Definitely would	37.5	0.010
	Possibly would	20.8	
	Probably not	20.8	
	Definitely not	20.8	

Bass was a strong advocate of unwaxed floss because he believed that wax might be left in the interproximal area which acts as an irritant. The greater thickness of the waxed floss tend to separate teeth slightly, resulting in soreness of gingival tissue.^14^ Carter et al. found the professional and self-administration of waxed floss produced 50.4% and 62.2%, respectively reductions in the number of initial bleeding areas, with unwaxed floss reductions of 74.8 and 11 were found. The authors concluded that unwaxed floss was “slightly” more effective than the waxed floss.^15^

Corby found that after a 2 week's study period of 12–21-year-old well–matched twins, tongue, and tooth brushing plus flossing significantly decreases the microbial species associated with dental caries and periodontal disease.^16^ Schonauer have attempted to compare the relative effectiveness of waxed versus unwaxed floss, but the results have not been conclusive.^17^

Gumchucks was introduced by Oral Wise Co. in 2014, they are the only and first flossing system of their kind. Like nunchucks miniature, they feature disposable tips that are equipped with a 3/4-inch piece of dental floss. Marginal proximal plaque index was taken as it records the interproximal plaque in all three segments of mesiobuccal, distobuccal and lingual.

A significant reduction was recorded in the plaque marginal index for intragroup comparison of gumchucks from baseline to 6 weeks as gumchucks’ unique two handle system increases control and dexterity allowing even the youngest children to easily make the “c” shape with the floss so the child can floss more frequently. Significant values were obtained in the reductions in the plaque index in the intergroup comparison from 0 to 6 weeks between gumchucks and unwaxed floss as frequency of flossing increased in the gumchucks group than the unwaxed floss and correct flossing needs making a “c” shape, to get beneath the gum line, gumchucks is one of the only tools.

When asked about the frequency of flossing, there was a marked increase in flossing in the gumchucks group than unwaxed floss as gumchucks gives children with limited dexterity, especially, handles that provide for the better control, keeps the floss taut for painless and smooth entry, and avoids cut off blood circulation from floss-wrapped fingertips. Many subjects said that they would prefer to buy gumchucks than unwaxed floss because they are available in attractive design and is easy to use.

## CONCLUSION

Gumchucks have a high efficacy of plaque removal as well as easy in use for children routinely. Also when surveyed a majority of the patient's preferred gumchucks if available in the stores.
